# Multiomic Mass Spectrometry Imaging to Advance Future Pathological Understanding of Ocular Disease

**DOI:** 10.3390/metabo12121239

**Published:** 2022-12-09

**Authors:** Joshua Millar, Ema Ozaki, Susan Campbell, Catherine Duckett, Sarah Doyle, Laura M. Cole

**Affiliations:** 1 Centre for Mass Spectrometry Imaging, Biomolecular Research Centre, Sheffield Hallam University, Sheffield S1 1WB, UK; 2 Immunobiology Research Group, Department of Clinical Medicine, Trinity College Institute of Neuroscience (TCIN), School of Medicine, Trinity College Dublin (TCD), D02 R590 Dublin 2, Ireland

**Keywords:** age-related macular degeneration, copper, LA-ICP-MSI, MALDI-MSI, multimodal, ocular disease, SARM1, zinc

## Abstract

Determining the locations of proteins within the eye thought to be involved in ocular pathogenesis is important to determine how best to target them for therapeutic benefits. However, immunohistochemistry is limited by the availability and specificity of antibodies. Additionally, the perceived role of both essential and non-essential metals within ocular tissue has been at the forefront of age-related macular degeneration (AMD) pathology for decades, yet even key metals such as copper and zinc have yet to have their roles deconvoluted. Here, mass spectrometry imaging (MSI) is employed to identify and spatially characterize both proteomic and metallomic species within ocular tissue to advance the application of a multiomic imaging methodology for the investigation of ocular diseases.

## 1. Introduction

Ocular tissue is a complex anatomical system constituted by multiple tissue types and organized in a highly structured and highly privileged manner. Disruption of this otherwise balanced structure can lead to the onset of ocular diseases that, without viable treatment, may lead to visual impairment [1]. Key to therapeutic discovery is a deeper understanding of the underlying mechanisms of disease onset. Age-related macular degeneration (AMD) is one of the most common ocular degenerative diseases, making up over 50% of legal blindness cases in England and Wales [2]. Currently, there are around 200 million patients worldwide, but incidence is expected to rise as populations begin to age [3]. There are presently very limited treatments for AMD, with only 15% of patients having viable treatment available to them [4,5,6]. This lack of viable therapeutics can be attributed to inadequate understanding of AMD pathology. Recent studies have linked a class of proteins called pattern recognition receptors (PRRs) and other proteins associated with the innate immune system in AMD pathology, implicating chronic inflammation and immune-mediated retinal damage in AMD [7,8]. Current immunological studies, however, have been limited by factors such as insufficient specificity, leading to disputes within the literature around the involvement of certain PRRs in AMD pathology [9]. Additionally, as highlighted in the Age-Related Eye Disease Study (AREDS) at the turn of the century, essential trace elements are crucial for regulation of inflammation and in the prevention of disease [10]. Following this, the National Eye Institute, began to recommend supplementation of zinc as a precautionary measure for those susceptible to AMD. Though copper was present to avoid hypocupremia, both zinc and copper have a role in the mitigation of inflammation as part of copper-zinc superoxide dismutase, and deficiencies in zinc can cause an inflammatory response [11]. The role of both essential and non-essential metals in ocular tissue in AMD has been associated with AMD onset.

Multiple MS approaches have been utilized in the past few decades to attempt to further the understanding of AMD etiology. MALDI-MS has been applied in the investigation of noninvasive tear analysis [12], proteomic changes in the aqueous humor and retina [13,14] as well as MSI of retinal lipids [15] in the context of AMD. Additionally, ICP-MS investigations into AMD tissue, conducted as early as 2000, have been seen to corroborate the findings within the AREDS [16,17]. Similarly, ICP-MS has mostly been applied to noninvasive matrices [16,17,18,19,20,21,22,23] or through analysis of the aqueous humor [24], with fewer targeting retinal tissue [17,25,26]. Invariably, few techniques utilize the multiplexing and multi-dimensional nature of 2D MS imaging, with even fewer studies combining techniques in a multimodal fashion [27]. 

## 2. Materials and Methods

### 2.1. Tissue Samples

Tissue was obtained from Trinity College Dublin (Dublin, Ireland) and consisted of fresh frozen C57Bl/6J wildtype mouse ocular tissues sectioned at 10 µm after being embedded in carboxymethylcellulose (CMC).

### 2.2. Chemicals

Ethanol (200 proof) from Honeywell (Bracknell, UK), glacial acetic acid (Fisher Scientific, Loughborough, UK), acetonitrile (Fisher Scientific, Loughborough, UK), octyl-α-β-glucoside (Sigma-Aldrich, Dorset, UK), trypsin (Promega, Southampton, UK), TFA (Sigma-Aldrich, Dorset, UK), CMC (Merck Group, Darmstadt, Germany), traceCERT^®^ Multielement Standard Solution 6 for ICP (Merck Group, Darmstadt, Germany), HNO_3_ (VWR, Poole, UK) and gelatin (Merck Group, Darmstadt, Germany) were used in this study.

### 2.3. MALDI-MSI Sample Preparation

#### 2.3.1. Sample Washing

Salts and lipids were removed using sequential washing with 70% EtOH, 90% EtOH, CHCl_3_ and 90:9:1 EtOH:AcOH:H_2_O for 1 min with drying steps in between.

#### 2.3.2. On-Tissue Digestion

First, 20 µg/mL of MS-grade trypsin (Promega, Southampton, UK) was prepared in 50 mM NH_4_HCO_3_ with 0.1% octyl-α-β-glucoside applied to the tissue via the use of sequencing grade trypsin. Then, 15 layers of 45 °C trypsin were applied at 10 psi N_2_ pressure and at 20 µLmin^−1^ using an HTX M3+ Sprayer (HTX Imaging, Chapel Hill, NC, USA). Following application, the samples were placed in a humidity chamber containing 50% methanol before incubation overnight at 37 °C. On removal, the samples were allowed to reach room temperature before matrix application.

#### 2.3.3. Matrix Application

A matrix consisting of 5 mgmL^−1^ α-cyano-hydroxycinnamic acid (CHCA) was prepared in a 50% acetonitrile solution with 0.01% TFA. It was then sonicated and syringe-filtered prior to application using an HTX M3+ (HTX Imaging, Chapel Hill, NC, USA) at 80 °C, using a solvent flow rate of 100 µLmin^−1^ and a N_2_ pressure of 10 psi, in 8 layers. Following matrix application, the samples were either used in MALDI analysis immediately or placed into slide mailers, vacuum sealed and stored at −80 °C for a maximum of 1 week. 

### 2.4. MALDI-MSI Analysis

All MALDI-MS analyses were conducted on two instruments: a Waters Corporation (Wilmslow, UK) SELECT SERIES MRT and a SYNAPT G2 HDMS. The SYNAPT was operated in sensitivity mode with positive polarity using a 1 kHz Nd:YAG laser at a power of 250 a.u. and a spot size of 50 µm. The acquisition mass range was 600–2500 Da. The MRT was operated in *MRT* mode, wherein the flight path was over 47 m and the resolution was ≥200,000 FWHM. The 2 kHz laser was operated at 1 kHz and was attenuated with 2 ND filters. The primary variable filter was applied at 300, and the secondary fixed filter was engaged. The laser was rastered over the sample at 125 µms^−1^, utilizing a 25 µm step size. A quad profile was set for optimum transmission of the analytes between 700 and 2500 Da, and the scan rate was set to 0.2 s, while data acquisition and processing were conducted using MassLynx version 4.2, HDI version 1.7, (Waters Corporation, Wilmslow, UK) and SCiLS Lab MVS version 2022b Pro (Bruker GmbH, Bremen Germany).

### 2.5. LA-ICP-MSI Sample Preparation

Prior to further analysis by LA-ICP-MS, the MALDI matrix was removed from the samples using a matrix wash step of 70% EtOH (2 × 1 min) before overnight desiccation.

#### LA-ICP-MSI Calibration Arrays

The calibration standards were prepared using an ICP Standard solution. Serially diluted standards were made in a range between 0–50 ppm by diluting in 1% HNO_3_, with an ^115^In internal standard at 1 ppm. Equal parts of each standard were then mixed with 20% (*w*/*v*) gelatin from porcine skin and vortexed before spotting 15 µL of each into 4 mm nylon wells. Once in the wells, the standards were allowed to dry overnight in a vacuum desiccator prior to analysis.

Gelatin from the porcine skin, carboxymethyl cellulose (CMC), ICP Standard Solution 6 (100 gL^−1^) and HNO_3_ (69%) were used in the preparation of the 1% CMC, 2.5% gelatin, 5% gelatin and 10% gelatin standards.

### 2.6. LA-ICP-MS Analysis

The LA-ICP-MS experiments were conducted on a UP-213 LA system (New Wave Research, Huntingdon, UK) and an ImageBio266 (Elemental Scientific Lasers, Inc. Huntingdon, UK) connected to a NexION 350x ICP-MS. Data were analyzed using Iolite version 4.4.6 (Elemental Scientific Lasers, Inc. Bozeman, MT, USA). The calibration arrays were analyzed prior to imaging and afterward, and they were processed to account for instrumental drift. Additionally, following quantitative imaging, the calibrants were analyzed by ICP-MS following a microwave digestion protocol to ascertain their true concentrations.

## 3. Results

### 3.1. Matrix Application

**Figure 1:**
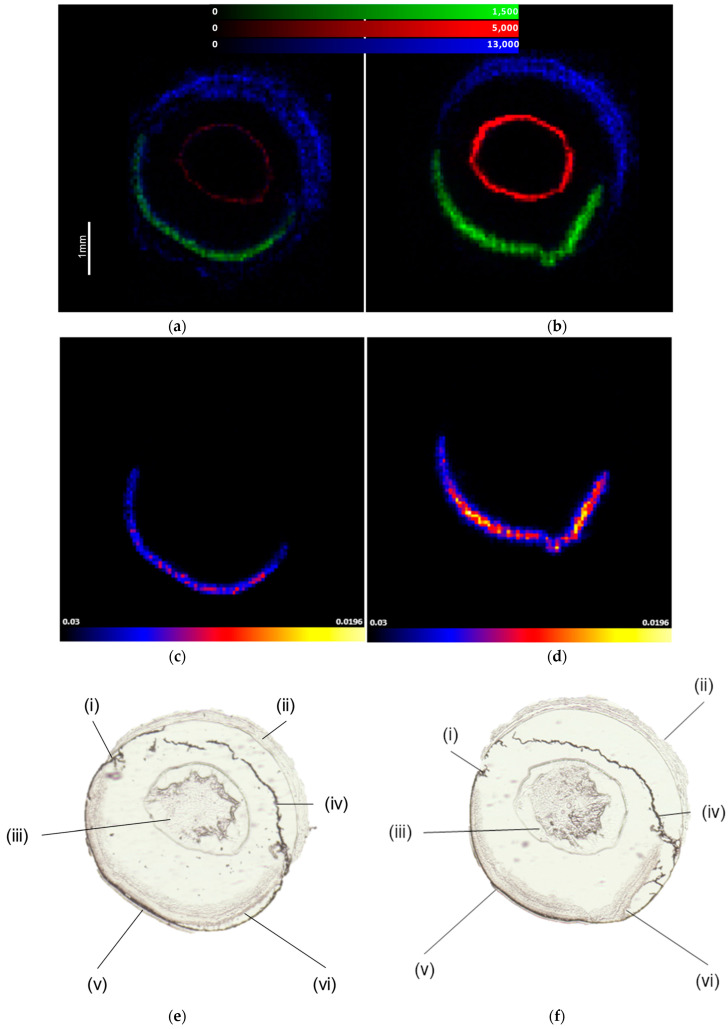
Images of 3 ions in the lens (*m*/*z* 1000.45), cornea (*m*/*z* 836.44) and retina (*m*/*z* 931.55), exhibiting the effects of different matrix application flow rates: (**a**) 50 µLmin^−1^ application with 3 peptide ions to contrast, (**b**) 75 µLmin^−1^ application with 3 ions to contrast, (**c**) 50 µLmin^−1^ application with 1 ion representing the retina and (**d**) 75 µLmin^−1^ application with 1 ion representing the retina. (**e**) The anatomy of the mouse ocular tissue (50 µLmin^−1^) and (**f**) the anatomy of the mouse ocular tissue (75 µLmin^−1^), with (i) ciliary body, (ii) cornea, (iii) lens, (iv) cris, (v) choroid and (vi) retina. Data acquired on a Waters SYNAPT G2 HDMS and processed using HDI version 1.7. Data from each dataset were normalized by TIC.

The MALDI-MSI images acquired of three ions using two different flow rates on the HTX M3+ sprayer can be seen in Figure 1. It can be seen from the images that the same ions in the serial sections of mouse ocular tissue increased in intensity when a higher flow rate of 75 µLmin^−1^ was used in comparison with a lower rate of 50 µLmin^−1^. Notably, despite the increase in flow rate, the ions within the MS image seemingly did not suffer from lateral delocalization within the retinal region, allowing better sensitivity within the experiments without compromising the spatial resolution.

### 3.2. MALDI-MSI Results

**Figure 2:**
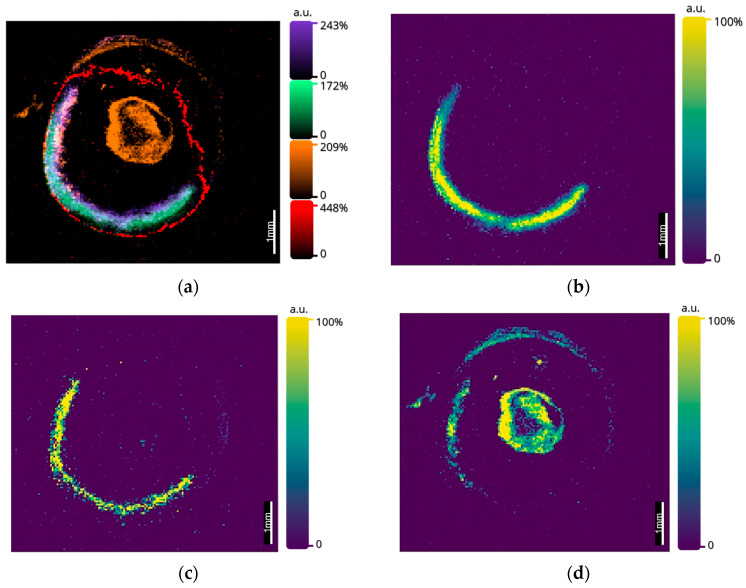
(**a**) An overlay image of 4 peptides within mouse ocular tissue, acquired by MALDI MS on a SELECT SERIES MRT at 25 µm with leptin *m*/*z* 1729.10 (red), lens crystallin *m*/*z* 1255.55 (orange), SARM1 *m*/*z* 1606.84 (green) and histone H32 *m*/*z* 1032.60 (purple). (**b**) SARM1 *m*/*z* 1606.84. (**c**) Histone H32 *m*/*z* 1032.60. (**d**) Lens crystallin *m*/*z* 1255.55. Data acquired on a Waters SELECT SERIES MRT.

Using the optimized matrix application methodology, Figure 2 shows images of SARM1 (*m*/*z* 1606.84 ± 1.12 ppm; see Table 1), an innate immune protein with pro-degenerative function known to be expressed mainly in photoreceptors and retinal ganglion cells [28]. The mass-to-charge ratios of SARM1 and other endogenous species such as histone H32, a common tryptic peptide observable in tissue, were tentatively identified using MALDI-MS at a mass resolution of 200,000 FWHM. SARM1 can be seen to localize within the retina, and when comparing to the ion image for histone H32 (*m*/*z* 1032.5975 ± 2.51 ppm; see Table 1), it is clear that the SARM1 peptide appeared to localize more specifically to the outer segment, outer nuclear area and chorio-retinal region, as opposed to histone H32, which localized more specifically to the inner nuclear layer and inner plexiform layer.

**Figure 3:**
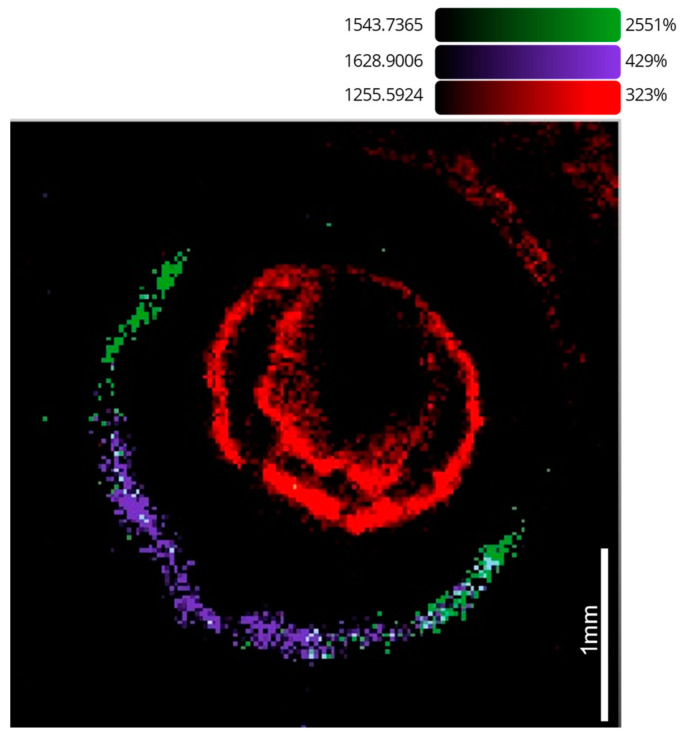
An overlay MALDI MS image acquired on the SELECT SERIES MRT at 25 µm, showing a lens crystallin *m*/*z* 1255.55 (red), proteasome assembly chaperone 4 (PSMG4) *m*/*z* 1543.73 in the anterior retina and CCS *m*/*z* 1628.90 in the posterior retina (purple). Data acquired on a Waters SELECT SERIES MRT.

Further images at 25 µm were once more able to segment the retinal substructure laterally, but rather than doing so in layers, as observed with SARM1 and histone H32 in Figure 2, through the observation of the copper chaperone for superoxide dismutase (CCS) and proteasome assembly chaperone 4 (PSMG4), MALDI-MSI was able to bisect the retina into anterior and posterior sections (Figure 3). This localization of ions suggests a higher relative intensity of CCS within the posterior region of the eye, meaning any copper observed within the retina may have been transported by CCS. Consequently, the copper observed throughout the rest of the ocular tissue may have been transported by different means, especially if observed in equal or greater quantities than the copper that co-localized with CCS.

### 3.3. LA-ICP-MSI Results

#### 3.3.1. Qualitative LA-ICP-MSI Results

**Figure 4:**
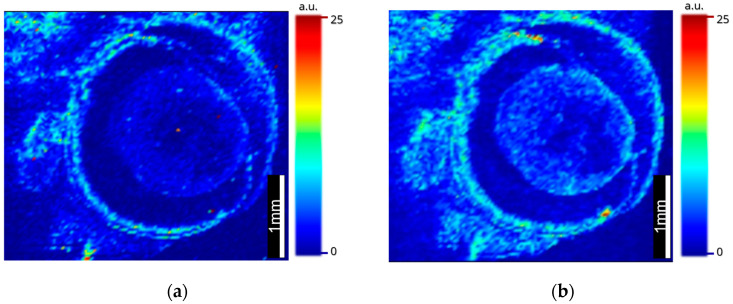
LA-ICP-MS images conducted at a 40 µm spot size on a UP-213 coupled to a NexION350x: (**a**) ^66^Zn in mouse ocular tissue and (**b**) ^63^Cu in mouse ocular tissue.

LA-ICP-MS was employed on sections previously examined by MALDI-MSI. Using two different LA units, a variety of spot sizes was utilized to produce ion images. Using an NWR UP-213 laser ablation unit, the 40 µm spot size images shown in Figure 4 were produced, which show that ^66^Zn and ^63^Cu were present throughout the ocular tissue, ^66^Zn was present in the cornea, lens retina and iris, and ^63^Cu shared a similar distribution, though it was seen to localize more predominantly in the ciliary body and on either side of the iris. 

**Figure 5:**
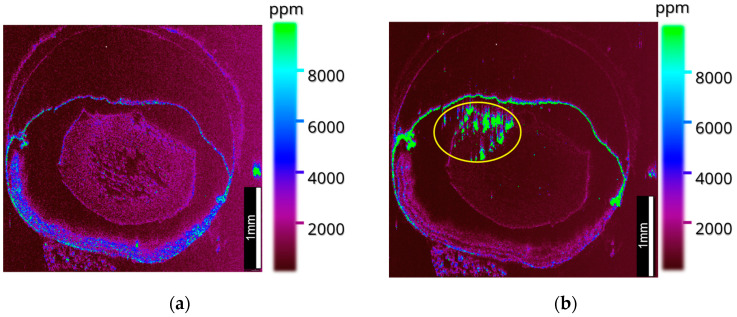

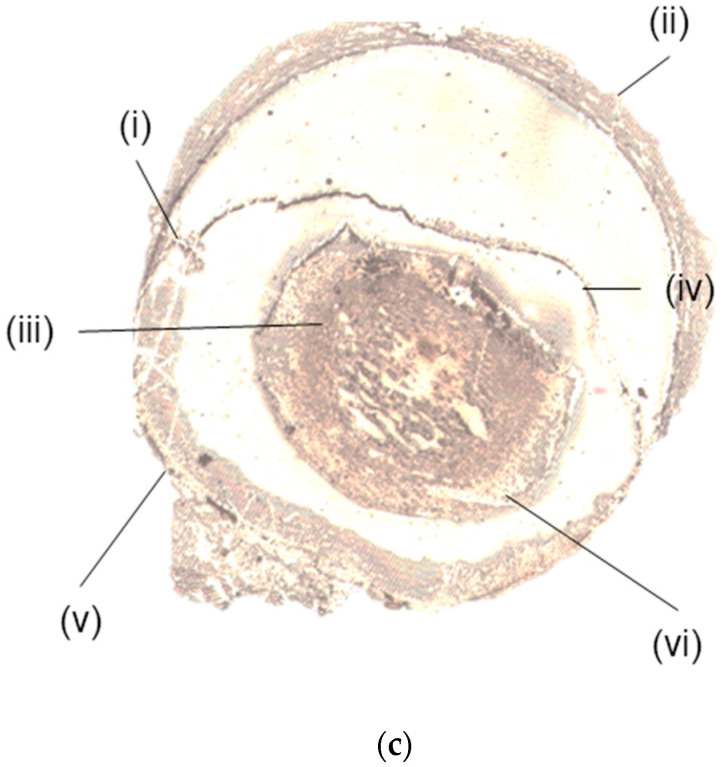
LA-ICP-MS images taken at 10 µm on an ESL ImageBio266 coupled to a NexION 350x: (**a**) ^66^Zn distribution within mouse ocular tissue and (**b**) ^63^Cu distribution within mouse ocular tissue N.B. The speckled region in the lens (circled in yellow) was identified as an artifact of the experiment and did not appear in subsequent experiments. (**c**) The anatomy of the mouse ocular tissue: (i) ciliary body, (ii) cornea, (iii) lens, (iv) iris, (v) choroid and (vi) retina.

Utilizing the faster washout times of the ESL ImageBio266, the 10 µm spot size images improved in spatial resolution in comparison with the 40 µm images while maintaining comparable acquisition times. The 10 µm images (Figure 5) show that the observations from the previous images could be corroborated, as ^66^Zn was observed in the retina and could be seen within the lens and iris. As in Figure 4, the ^63^Cu can be seen to localize most predominantly in the ciliary body, though this image also shows larger amounts within the iris.

#### 3.3.2. Quantitative LA-ICP-MSI Results

Quantitative ICP-MS was employed in order to add further dimensions to the data. One of the key aspects of this approach is the preparation of reproducible calibration arrays. Table 2 shows how inconsistencies appeared when initially trying to prepare the calibration arrays, as there were statistically significant differences in the repeats of the same elements.

**Figure 6:**
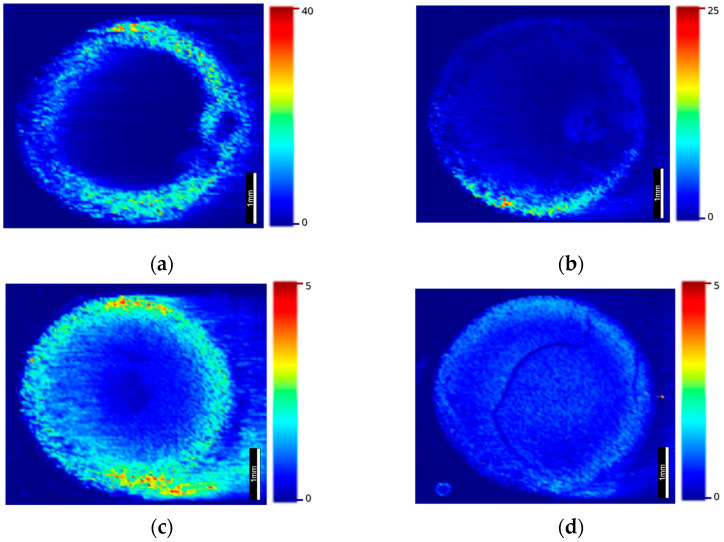
LA-ICP-MS images showing the spatial distribution of zinc in calibration arrays made from (**a**) 1% CMC, (**b**) 2.5% gelatin, (**c**) 5% gelatin and (**d**) 10% gelatin.

In order to ascertain the source of these anomalies in the quantitative calibration arrays, images were taken on the UP-213 at 40 µm to see if the calibration arrays were homogenous. Figure 6a shows how zinc was not being deposited homogenously. Figure 6b–d exhibits how this Marangoni effect can be mitigated through the use of low aqueous calibration arrays.

**Figure 7:**
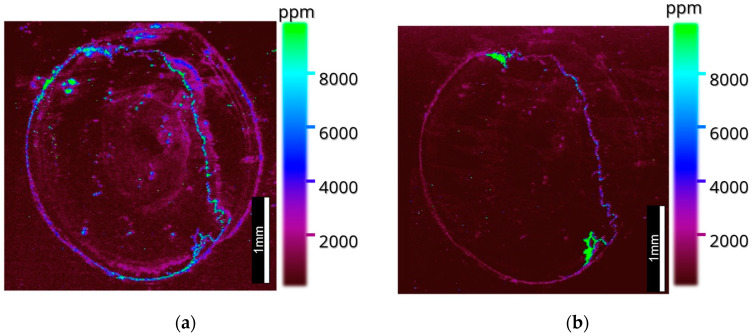
Quantitative LA-ICP-MS images taken at 10 µm on an ESL ImageBio266 coupled to a NexION 350x: (**a**) ^66^Zn distribution within mouse ocular tissue and (**b**) ^63^Cu distribution within mouse ocular tissue.

The images produced with the optimized calibration arrays (Figure 7) were similar to those in Figure 5, showing the localization of ^66^Zn in the retina, lens and iris and ^63^Cu again localizing within the ciliary body, with smaller amounts in the iris. With the added dimension of quantitative data, analysis showed that ^66^Zn was localized in higher quantities than ^63^Cu within the lens, cornea and choroid, whereas ^63^Cu was observed in higher quantities within the ciliary body of the ocular tissue and in similar quantities to ^66^Zn within the iris.

## 4. Discussion

### 4.1. MALDI Sample Preparation Optimization

To ensure the deposition method provides enough spatial resolution, this is most often determined by the crystal size of the matrix, which is heavily dependent on the application methodology [29]. Additionally, the lateral migration of the proteins must be limited, which can happen when a tissue becomes overly wet or exposed to high volumes of an organic matrix [30]. Generally, with an optimal matrix solvent, matrix concentration and gas flow, robotic sprayers can provide relatively small crystal sizes of around 5–25 µm [31]. The data provided in Figure 1 show the benefit of higher flow rates while simultaneously exhibiting the value of robotic sprayers such as the HTX M3+ and their ability to provide heated solvent deposition, which simultaneously helps reduce the matrix crystal size by allowing high organic matrix solvent composition while preventing lateral delocalization.

### 4.2. MALDI-MSI

SARM1 has previously been identified as a regulator of crucial biochemical processes within axonal degeneration, a process key to a variety of retinal degenerative diseases whereby SARM1 regulates the depletion of essential metabolites (NAD) and induces an energy crisis [28,32,33]. Thus, a deeper understanding of the biology of SARM1 within healthy and diseased tissue could prove to be pivotal in finding treatment for retinal degenerative diseases in the future (e.g., SARM1 inhibitors). Here, SARM1 can be seen to localize within the posterior region of the retina, namely the outer nuclear layer adjacent to the RPE and choroid, a region which is key in the onset of both wet and dry AMD [5] and which is supportive of previous localization data for this protein [28].

Additionally, peptides related to the transport and regulation of metals within the retina were observed. Figure 3 shows the observation of CCS within the posterior region of the ocular tissue. The ability to image proteins related to the regulation and transport of key trace elements may in the future provide unique insight into the role of accumulating essential and non-essential trace metals within diseased ocular tissue, helping to further understand the co-dependencies that exist in the proteomic and metallomic changes that occur in ocular disease. The mapping of metals as well as the proteins by which they are transported is of great benefit, utilizing a multifaceted and multimodal approach.

### 4.3. LA-ICP-MSI

#### 4.3.1. Qualitative Imaging

Previous studies that have investigated the accumulation or relation of trace metals to AMD have been both qualitative and quantitative, but fewer have included the added dimension that imaging data can provide to this niche of pathobiology [17,18,19,25]. Figure 4 shows that zinc and copper were distributed within the choroid and less so in the lens, cornea and iris. These data not only corroborate past reports that cite increased levels of metal within the choroid and RPE but add a further dimension, showing that the metals were evenly distributed throughout the choroid.

The 10 µm images produced in Figure 5 corroborated the findings from the previous images, exhibiting the localization of copper to the ciliary body and choroid [16]. The presence of copper within the ciliary body was most likely associated with the high levels of copper found within muscle tissue, though as the ciliary body is responsible for the production of aqueous humor, and AMD patients are often seen to have a copper deficiency in their aqueous humor, this may prove to be an interesting insight in the future [17,18,19,24,25,34]. Additionally, copper was seen to localize to the exterior regions of the lens, which constitutes the youngest part of the lens [34]. The observation of copper and zinc within the choroid was, however, most important, as it has previously been reported that this area of the ocular structure is subject to the most change in the elderly. Wills et al. reported a significant increase in copper and zinc in the choroid but a reduction within the neural retina in aged ocular tissue when compared with younger tissue [16]. Erie et al. further demonstrated the changes that occur in the choroid by showing that of 44 participants, those with AMD had significantly lower amounts of copper and zinc within their choroids, which aligns with the advice provided by the AREDS [10,17].

#### 4.3.2. Quantitative Imaging

While quantitative data have provided key information about the wider mechanisms of essential metals within ocular physiology, quantitative ICP is not without its limitations. The reliability of quantitative conclusions drawn from ICP experiments is susceptible to elemental fractionation, a phenomenon that encompasses the effects of the perceived compositions of ICP samples caused by factors such as the preferred ablation of more volatile compounds, the time-dependent changes in the ion beam and the transport efficiency of differently sized aerosol particles [35]. Overcoming these issues can be fundamental in the success of a quantitative study on biological samples. However, the introduction of imaging, and therefore the use of laser ablation, involves additional factors affecting quantitative analysis. The main issue stemming from laser ablation is the effects of the matrix [35,36].

The problem becomes most pertinent when using quantitative standards, as different matrices will, for example, behave differently when subjected to an incident laser beam and will thereby produce aerosols more or less efficiently, depending on properties such as the thermal conductivity, absorptivity and reflectivity [35]. As a consequence, when using quantitative standards, the matrix chosen to hold the elemental standards must behave in an analogous manner to the analyte. Gelatin, agarose gel or sol-gel in the past have been the most common matrices for elemental standards used to mimic the carbon content and density of the biological sample without the need for expensive CRMs [35,36,37]. Herein, the use of gelatin, in addition to an MS-friendly medium of carboxymethyl cellulose, were trialed as potential matrices to house the elemental standards for quantitative ICP imaging.

We prepared and mixed 1% CMC and 5%,10% and 20% gelatin in equal parts with 50 ppm of ICP Solution 6, yielding m/v values of 0.5%, 2.5%, 5% and 10%, respectively. The standards were first prepared by spotting 15 µL into M4 metric washers and dried in a vacuum desiccator overnight at room temperature. Regarding the utilized parameters optimized for mouse ocular tissue, the LA standards were acquired by using one line per standard. Using the average intensities from the line scans, calibration curves were produced. The data showed poor regressions (Table 2).

To identify the source of the anomalous regressions given to the samples, images of the gelatin spots were taken to ascertain if uneven distributions were the cause of the anomalous results. The images were taken at 40 µm. The results (Figure 6) showed that the CMC, 2.5% gelatin and 5% gelatin were exhibiting characteristics of the Marangoni effect and producing rings of high concentrations at the periphery of the standards. To avoid this, lower aqueous standards were prepared which, as demonstrated by Šala et al., could help reduce the effects caused by capillary action, as demonstrated in Figure 6d [36]. Then, 10% gelatin was used thereafter within the LA standards which, when acquired alongside an image of mouse ocular tissue at 10 µm (Figure 7), were able to produce a quantitative LA-ICP-MS image. The images were able to inform of the previously viewed data, which showed what appeared to be lower levels of zinc within the sclera, but by normalizing the scales of copper and zinc to one another, it can be seen that zinc was in higher quantities in the choroid when compared with copper, concurring with previous studies that employed excision as a way to quantify the metal content within the choroid [16].

The distribution of zinc in ocular tissue is poorly understood in the context of AMD, yet zinc deficiency is one of the hallmarks of AMD and has been attributed to the onset of AMD in later life [10]. Additionally, zinc deficiency is seen to be inflammatory through the induction of IL6 promoter demethylation. ^11^ Whilst AREDS recognized the importance of zinc deficiency in AMD onset, and the links between zinc and the inflammatory response are well documented, little work has been performed that explores a potential synergy between the proteomic and metallomic changes in the aging eye that may result in chronic inflammation. In Figure 5 and Figure 7, there is a clear demarcation between the metal distribution within the choroid in the outer blood–retinal vascular bed and the inner retina as well as distinct proteomic segments, exhibiting the similar behavior of metals and proteins within the highly privileged retinal structure [1].

In summary, the combination of both proteomic analysis by MALDI-MSI and metal analysis by LA-ICP-MSI exhibited the utility of applying the extra dimension of 2D qualitative and quantitative imaging to complex heterogenous ocular tissue. Additionally, the multiplexing nature of the experiments that can be conducted by using these imaging techniques in tandem as part of a multiomic approach was demonstrated. By using a workflow that not only informs of the proteomic changes within a tissue but also the metallomic changes, a broader understanding of the underlying mechanisms of ocular disease can be achieved.

## 5. Conclusions

The workflow described here invites an opportunity to alter the way in which mass spectrometry is used to study ocular disease. Current methodologies for studying ocular diseases such as AMD risk stagnation without an interdisciplinary and multidimensional approach to their investigation. Studies on the AMD proteome by immunological methods could benefit from complimentary mass spectrometry data and yet further improved with the added dimension of mass spectrometry imaging. Likewise, studies into the essential metals related to AMD stand to gain from the use of quantitative LA-ICP-MS imaging to not only quantify the changes in metal content in healthy and AMD eyes but map the changes of distribution within the ocular tissue in relation to the proteomic changes that occur within the same space in combination with MALDI-MSI. However, though mouse models have long been used for the modeling of disease due to convenience and economy, mouse models offer issues, as they are nocturnal creatures with ocular adaptations different to humans and additionally do not possess a macula [38,39]. As a result, to further the significance of these studies, future work will include investigations of the localization of the aforementioned classes of proteins within the human retina. When used in tandem as described here, these imaging techniques, when applied to diseased ocular tissue, offer multi-dimensional and multifaceted data from a single tissue section, and if applied in an inter-disciplinary manner, they will aid the development of research on a variety of ocular diseases. In addition, abbreviations used here are extensively listed for clearer understanding in Abbreviations.

## Figures and Tables

**Table 1:** Relative error values for those ions observed in Figure 2.

ID	ppm	m_exp_	m_calc_	Position	No. of Missed Cleavages
Histone H32	2.51	1032.5975	1032.5949	42–50	0
Lens crystallin	3.02	1255.5487	1255.5429	835–845	0
SARM1	1.12	1606.8428	1606.8410	202–216	0
Leptin	1.44	1729.9963	1729.9938	27–41	2

**Table 2:** Regression figures given by calibration arrays prepared in carboxymethyl cellulose and gelatin from porcine skin.

Element	CMC	Gelatin (5%)	Gelatin (10%)
^63^Cu	0.9574	0.9776	0.9939
^66^Zn	0.9438	0.9696	0.9637

## Data Availability

The data presented in this study will be made available at the Sheffield Hallam University Research Data Archive (SHURDA) and will be found at https://shurda.shu.ac.uk. (accessed on 1 December 2022).

## Abbreviation

**Abbreviation**	**Definition**
AMD	Age-Related Macular Degeneration
AREDS	Age Related Eye Disease Study
CCS	Copper Chaperone for Superoxide Dismutase
CHCA	A-Cyano-4-Hydroxycinnamic Acid
CMC	Carboxymethylcellulose
FWHM	Full Width at Half Maximum
ICP	Inductively Coupled Plasma
LA	Laser Ablation
MALDI	Matrix Assistance Laser Desorption Ionisation
MRT	Multi-Reflecting Time-Of-Flight
MS	Mass Spectrometry
MSI	Mass Spectrometry Imaging
NAD	Nicotinamide Adenine Dinucleotide
ND	Neutral Density
Nd:YAG	Neodymium-Doped Yttrium Aluminium Garnet
NIH	National Institutes of Health
ppm	Parts Per Million
PRRs	Patter Recognition Receptors
PSMG4	Proteasome Assembly Chaperone 4
SARM1	Sterile Alpha and TIR Motif Containing 1
